# Bayesian model selection techniques as decision support for shaping a statistical analysis plan of a clinical trial: An example from a vertigo phase III study with longitudinal count data as primary endpoint

**DOI:** 10.1186/1471-2288-12-137

**Published:** 2012-09-10

**Authors:** Christine Adrion, Ulrich Mansmann

**Affiliations:** 1 Institute for Medical Information Sciences, Biometry and Epidemiology (IBE), Ludwig-Maximilians University, 81377 Munich, Marchioninistr. 15, Germany

**Keywords:** Statistical analysis plan, Sensitivity analysis, Longitudinal count data, Bayesian generalized linear mixed models, INLA, Predictive performance, Bayesian model evaluation, Informed model choice

## Abstract

**Background:**

A statistical analysis plan (SAP) is a critical link between how a clinical trial is conducted and the clinical study report. To secure objective study results, regulatory bodies expect that the SAP will meet requirements in pre-specifying inferential analyses and other important statistical techniques. To write a good SAP for model-based sensitivity and ancillary analyses involves non-trivial decisions on and justification of many aspects of the chosen setting. In particular, trials with longitudinal count data as primary endpoints pose challenges for model choice and model validation. In the random effects setting, frequentist strategies for model assessment and model diagnosis are complex and not easily implemented and have several limitations. Therefore, it is of interest to explore Bayesian alternatives which provide the needed decision support to finalize a SAP.

**Methods:**

We focus on generalized linear mixed models (GLMMs) for the analysis of longitudinal count data. A series of distributions with over- and under-dispersion is considered. Additionally, the structure of the variance components is modified. We perform a simulation study to investigate the discriminatory power of Bayesian tools for model criticism in different scenarios derived from the model setting. We apply the findings to the data from an open clinical trial on vertigo attacks. These data are seen as pilot data for an ongoing phase III trial. To fit GLMMs we use a novel Bayesian computational approach based on integrated nested Laplace approximations (INLAs). The INLA methodology enables the direct computation of leave-one-out predictive distributions. These distributions are crucial for Bayesian model assessment. We evaluate competing GLMMs for longitudinal count data according to the deviance information criterion (DIC) or probability integral transform (PIT), and by using proper scoring rules (e.g. the logarithmic score).

**Results:**

The instruments under study provide excellent tools for preparing decisions within the SAP in a transparent way when structuring the primary analysis, sensitivity or ancillary analyses, and specific analyses for secondary endpoints. The mean logarithmic score and DIC discriminate well between different model scenarios. It becomes obvious that the naive choice of a conventional random effects Poisson model is often inappropriate for real-life count data. The findings are used to specify an appropriate mixed model employed in the sensitivity analyses of an ongoing phase III trial.

**Conclusions:**

The proposed Bayesian methods are not only appealing for inference but notably provide a sophisticated insight into different aspects of model performance, such as forecast verification or calibration checks, and can be applied within the model selection process. The mean of the logarithmic score is a robust tool for model ranking and is not sensitive to sample size. Therefore, these Bayesian model selection techniques offer helpful decision support for shaping sensitivity and ancillary analyses in a statistical analysis plan of a clinical trial with longitudinal count data as the primary endpoint.

## Background

A statistical analysis plan (SAP) is a critical link between how a clinical trial is conducted and the clinical study report. To secure objective study results, regulatory bodies expect that the SAP will meet requirements in pre-specifying inferential analyses and other important statistical techniques. Writing a good SAP for a model-based sensitivity or ancillary analysis [1,2] involves non-trivial decisions on and justification of many aspects of the chosen model setting. In particular, trials with longitudinal count data as primary endpoint pose challenges for model choice and model validation. This paper explores tools for this decision process when sensitivity analyses are performed using generalized linear mixed models (GLMMs) for the analysis of longitudinal count data. These tools can be used to build transparent strategies for shaping the final models reported in the SAP.

The documentation of longitudinal profiles for the primary endpoint offers many advantages. They are more informative compared with a single timepoint analysis and give information about the ’speed of efficacy’ [3]. Treatment effects evaluated by comparing change over time in quantitative outcome variables between the treatment groups are of great interest [4,5]. The analysis of longitudinal profiles offers an effective way to handle composite endpoints like: (1.) the long-term effect of experimental treatment (E) is better than that of standard treatment (S), and (2.) patients under E reach a pre-specified effect faster than those under S.

We are interested in parametric modeling approaches for quantifying absolute effects, adjusting for baseline covariates and handling stratification. There is a rich literature on nonparametric methods for longitudinal data, for example, Brunner et al. [6]. These models do, in general, allow estimation of relative effects. Omar *et al.*[7] provide an overview of several alternative parametric approaches in trying to deal with individual longitudinal profiles: (i) the ’summary statistic method’ [8] using a suitable summary measure (e.g. rates of change, post-treatment mean, last value of the outcome measure, or area under a curve) calculated for each subject, and subsequently analyzed with rather simple statistical techniques; (ii) repeated measures analysis of variance; (iii) marginal models based on generalized estimating equations (GEE) [9]; (iv) mixed effects modeling approach involving fixed and random effects components [10,11].

Mixed effects (or random effects) models allow us to investigate the profile of individual patients, estimate patient effects and describe the heterogeneity of treatment effects over individual patients. They account for different sources of variation (patient effects, center effects, measurement errors) and provide direct estimates of the variance components which might be of interest in their own right. Furthermore, they allow us to address various covariance structures and are useful for accommodating overdispersion often observed among count response data [10-12].

The EMA Guideline on Missing Data in Confirmatory Clinical Trials from 2010 [13] explicitly considers random effects approaches (i.e. generalized linear mixed effects models (GLMMs) in the case of a non-Gaussian response) as an approach to handling trials with a series of primary endpoints measured repeatedly over time. Mixed models are also helpful for handling missing values. They are applicable under missing completely at random (MCAR) as well as missing at random (MAR) [14], while simple repeated univariate analyses for each time point using test procedures such as the *t*-test, ANOVA, or the Wilcoxon rank sum test rely on the more restrictive assumption of MCAR. Also, for non-ignorable missing data mechanisms, newer model-based strategies for longitudinal analyses are increasingly available and offer the opportunity to account for dropout patterns (e.g. pattern mixture models [15]). To be fully compatible with the intention-to-treat (ITT) principle, one has to explicitly consider incomplete individual profiles to correctly incorporate the information available for all randomized patients.

These points in summary may explain why our interest focuses on GLMMs as a powerful tool for the sensitivity analysis of longitudinal count data. What we need is to pre-specify in detail a robust, valid, and parsimonious strategy for the data to come (see ICH E9, EMA or PSI Guidelines [13,16,17]). Writing the SAP prospectively for a randomized clinical trial with longitudinal *counts* as the primary endpoint asks for a series of decisions when specifying a GLMM for the analysis. Consideration should mainly be given to the following issues: 

*Distributional assumptions*: Poisson, negative binomial, or more sophisticated extensions, e.g., accounting for zero-inflation.

*Transformation* of outcome variable: e.g. log-transformation for skewed continuous positive variables [18], or variance-stabilizing transformations (e.g. inverse hyperbolic sine-transformation for non-negative count variables). An FDA guideline [19] postulates that a rationale for the choice of data transformation along with the interpretation of the estimates of treatment effects based on the transformed scale should be provided. In some situations, transformation of endpoint data is indicated and preferred to untransformed analyses on the original scale. However, careful consideration should be given to using a transformation which should be pre-specified at the protocol design stage.

*Variance-covariance structure*: specifying whether random effects (e.g. patient-specific intercept, patient-specific slopes) are appropriate; specification of the within-error structure. Altogether, random effects selection can be challenging, particularly when the outcome distribution is not normal (see [20-22] for more details).

*Methods for handling dropouts*: e.g. dealing with informative drop-outs, applying an analysis in which the last observation is carried forward, accounting for non-ignorable missing data mechanisms (pattern-mixture models). This approach must be fully compatible with the intention-to-treat principle.

*Use of covariate- or baseline-adjusted analyses*, handling multi-center data: specifying the mean structure by identifying the fixed effects terms.

The last issue is proposed by Pocock *et al.*[23] for avoiding misuse and data-driven selection of covariates within the clinical trial setting. The typical strategy for settling this complex issue is to decide on a simple model on which the primary analysis is based and to use sensitivity analyses to assess the robustness of the derived result under realistic model deviations.

In this paper, we propose using pilot or pre-study data to make an *informed choice* about the sensitivity analysis stated in the SAP. Pilot or pre-study (commonly called a “feasibility” or “vanguard” study) data come from a trial in an earlier phase, from a registry, or from a proof-of-concept study. For phase III trials, data from phase II trials generally exist [24]. In this respect we could also use data from the comparable treatment arms of related studies. Using these data helps to shape and justify *in advance* the modeling strategy for analyzing the main trial data, and to check the validity and the appropriateness of several model assumptions. It is imperative to minimize misspecification of the assumed GLMM, and this analysis enables the trial statistician to define a broad and robust setting for the final choice of the model.

Having determined the main focus of this paper, we need to motivate our choice of *Bayesian* tools for achieving our goal. Within the GLMM framework, analytical methods for model assessment and goodness-of-fit criteria are not straightforward, and *frequentist* approaches remain limited. The inclusion of random effects makes theoretical derivations rather complex, imposing computational challenges. Some proposed model evaluation procedures focus on checking the deterministic components (i.e. mean and variance-covariance structure) of a GLMM based on the cumulative sums of residuals, or assess the overall adequacy by means of a goodness-of-fit statistic which can be used in a manner similar to the well-known *R*^2^ criterion [25,26]. Furthermore, for small sample sizes, likelihood-based inference via penalized quasi-likelihood in the case of a longitudinal discrete outcome can be unreliable with variance components being difficult to estimate. In contrast, many easy-to-implement tools are available within the *Bayesian* framework. We will briefly review Bayesian tools developed recently and demonstrate their usefulness: For assessing goodness-of-fit and performing model validation, we apply the probability integral transform (PIT) [27-29] as a graphical posterior model check. We implement formal statistical criteria such as the deviance information criterion (DIC) [30], conditional predictive ordinate (CPO) [31,32], or proper scoring rules [28,29,33-36] to compare the forecasting capability of different competing GLMMs. A further objective is exploring the behavior of these different Bayesian methods for model validation concerning the coherence of their preference for a certain candidate model.

The article is organized as follows: The Methods section reviews Bayesian strategies for GLMMs in the count response situation. The main idea of integrated nested Laplace approximation (INLA) proposed by Rue *et al.*[37] is described briefly. We also introduce tools for model ranking and for evaluating the performance of the proposed model alternatives based on a *prediction-oriented* approach. Additionally, a case study is presented which will be used in the subsequent section to motivate the methodology. The Results section applies the proposed Bayesian methodology to clinical trial data on vertigo attacks and presents the findings of our simulation study. The Discussion section contains the limitations of the methods proposed. More technical material is provided in the Appendix. Selected R‐INLA code with further details concerning the INLA approach is included in the Web Supplementary Material of this paper [see Additional files 1 and 2.

For data analysis, inla-program [38] based on the open-source software R version 2.12.1 [39] was used to demonstrate the applicability of the Bayesian toolbox.

## Methods

### Bayesian generalized linear mixed models for longitudinal count data

In the following, regression approaches to modeling discrete count outcomes are briefly outlined. In the clinical trial setting, we assume that each patient *i*, *i* = 1,…,*N*, is randomized to a new drug (*x*_*i*_ = 1) or a standard treatment (*x*_*i*_ = 0). The observations *y*_*ij*_for each patient are counts measured in the course of time during each study visit, *j* = 1,…,*n*_*i*_ (presuming an imbalanced design), with time $\left(t_{\mathrm{ij}}\in R\right)$ and *t*_*i*1_ = 0. The linear predictor is defined as

(1) $$\eta_{\mathrm{ij}}=(\beta_{0}+b_{0i})+(\beta_{1}+b_{1i})\;t_{\mathrm{ij}}+\beta_{2}\;x_{i}+\beta_{3}\;x_{i}\;t_{\mathrm{ij}}\;,$$

with ***β***=(*β*_0_,*β*_1_,*β*_2_,*β*_3_)^T^being the *population-level* parameter vector (fixed effects), *b*_0*i*_ denoting patient-specific random intercepts and *b*_1*i*_subject-by-visit random slopes. The fixed effects (in a frequentist framework) account for group-specific effects (e.g. treatment group or time), serving at the same time as parameters of interest in a clinical trial. We want to relate the count response to explanatory variables such as time and treatment. In the most general case, a standard assumption for a GLMM with both random intercept and slope is that ***b***_*i*_=(*b*_0*i*_,*b*_1*i*_)^T^follows a bivariate normal distribution with mean zero and an unknown precision matrix **Q**=**Q**(***ϕ***) depending on parameters ***ϕ***, i.e.

$$b_{i}|Q{∼}_{\text{iid}}N_{2}(0,Q^{-1}).$$

The variance covariance matrix **Q**^−1^for variance components ***ϕ***is parameterized in terms of precisions and a correlation parameter. That is,

$$Q^{-1}=\left(\begin{array}{c} \;1/\tau_{b_{0i}}\;\;\;\rho /\sqrt{\tau_{b_{0i}}\tau_{b_{1i}}}\\ \rho /\sqrt{\tau_{b_{0i}}\tau_{b_{1i}}}\;\;1/\tau_{b_{1i}} \end{array}\right),$$

where *τ*_._ refers to the marginal precision of random effects *b*_*.i*_. Therefore, it is necessary to allow for the correlation *ρ*between random intercepts and slopes. In GLMMs formulated within a Bayesian framework, a non-Gaussian hyperprior distribution must be assigned to the precision matrix **Q**(***ϕ***), where *τ*_._ and *ρ*represent the hyperparameters. As proposed by Fong *et al.*[40] and Wakefield [41], we assume

$$Q∼{\text{Wishart}}_{2}(r,R^{-1}).$$

The prior parameters of the Wishart prior are (*r*,*R*_11_,*R*_22_,*R*_12_), where *r*>1 (to obtain a proper prior) in the case of two dependent random effects. *R*_12_ is element (1,2) of the inverse of **R** and *R*_12_ = *R*_21_ because of symmetry. Integration over **Q** gives a marginal *t*_2_(**0**,[(*r*−1)]^−1^**R**,*r*−1)-distribution of ***b***_*i*_=(*b*_0*i*_,*b*_1*i*_)^T^, with *t*_2_ denoting the Student’s *t* distribution with 2 degrees of freedom.

#### Poisson GLMM

Poisson loglinear regression is a common choice for modeling count response data. The probability function can be expressed as

$$\text{Pr}{\;}_{\mu }(y)=\exp(-\mu )\mu^{y}/y!$$

for *y* = 0,1,2,… and *μ*>0. For longitudinal count data with *i* = 1,…,*N* subjects and *j* = 1,…,*n*_*i*_ measurements per subject, the observed counts *y*_*ij*_ are conditionally independent Poisson variables *Y*_*ij*_∼ Poi(*μ*_*ij*_), with the conditional mean of *Y*_*ij*_ related to the linear predictor by a logarithmic link function. Let *μ*_*ij*_ = E(*Y*_*ij*_|***β***,***b***_*i*_). Hence, the resulting predictor in a standard Poisson GLMM for predicting the mean rate is

$$\log(\mu_{i})=\eta_{i}=X_{i}\beta +Z_{i}b_{i},$$

where **X**_*i*_ is an *n*_*i*_×*p* matrix and **Z**_*i*_ is an *n*_*i*_×*q* matrix, with ***β*** a *p*×1 vector of population-level parameters (fixed effects) and ***b***_*i*_a *q*×1 vector of zero-mean normally distributed random effects. In the longitudinal setting described in equation (1), *p* = 4, *q* = 2 and **Z**_*i*_ = (**1**,***t***_*i*_). The primary Poisson assumption is equidispersion, i.e. the equality of the mean and the variance functions. However, this is often inconsistent with empirical evidence. In reality, the value of the variance often exceeds that of the mean *μ*_*ij*_, resulting in overdispersion. Thus, although they are widely used to model count data, Poisson GLMMs may not well be suited to types of count outcomes from specific applications.

#### Negative binomial GLMM

A conventional modeling approach for *overdispersed* count data where the variance exceeds the mean (i.e. a given rate *μ*_*ij*_) is the negative binomial (NB) loglinear regression. In the classical univariate setting (dropping the subscript *i*), the NB density can be written as

$$\text{Pr}{\;}_{k,p}(y)=\frac{\Gamma (y+k)}{\Gamma (k)\;\Gamma (y+1)}p^{k}\;{\left(1-p\right)}^{y}\;,$$

for *y* = 0,1,2,…, probability 0<*p*≤1, and $\left(k\in R\right)$, *k*>0. *Γ*(*n*)=(*n*−1)! denotes the Gamma function, and *y* represents the number of failures which occur in a sequence of Bernoulli trials before a target number of successes is reached. Additionally, the hyperparameter *k* (often called “size”) plays the role of an overdispersion parameter. For negative binomial data, the corresponding mean and variance are then given by

$$\mu =\frac{k\;(1-p)}{p}\;\text{and}\;\sigma^{2}=\mu +\mu^{2}/k=\frac{k\;(1-p)}{p^{2}},$$

with *p*=*k*/(*k* + *μ*)=*μ*/*σ*^2^, *k*=*μ*^2^/(*σ*^2^−*μ*).

Overdispersion in the negative binomial model can be interpreted by unobserved heterogeneity among the observations *y*. If this phenomenon is not taken into account in the modeling process, it can lead to underestimated variance which then leads to incorrect posterior inference. It must be kept in mind that in the NB regression, the dispersion parameter takes observation-specific values. In the limit *k*→*∞*, holding *μ*fixed, the variance approaches the mean and therefore the negative binomial NB(*k*,*p*) converges to Poi(*μ*) (with $\left(\mu =k\;\frac{1-p}{p}\right)$) in a distributional manner.

#### Zero-inflated GLMM

In many biometrical and epidemiological applications, the count data encountered often contain a high proportion of extra zeros relative to the Poisson distribution, which is routinely applied for these situations. Therefore, a major source of overdispersion in these situations is a preponderance of zero counts. *Zero-inflated* count models provide a parsimonious yet powerful way to model this type of situation. Such models assume that the data originate from a mixture of two separate processes: one generates only zeros, and the other is either a Poisson or a negative binomial data-generating process. The result of a Bernoulli trial is used to determine which of the two processes generates an observation.

Hence, as regards zero-inflated estimation method in general, two regression equations are created: one predicting whether the count occurs (“always zero group”) and a second predicting differences in the occurrence of the count (“not always zero group”). While these differences are not modeled with standard Poisson or negative binomial regression, zero-inflated models first account for the excessive zeros by predicting group membership – i.e. an unobserved latent dichotomous outcome – based on the constellation of covariates included in the model and then predicting frequency of counts for only those in the “not always zero group”. The zero-inflated version of a distribution *D* of a random variable *Y*∼ZID(*Π*_0_,***θ***), where ZID denotes a zero-inflated distribution, has a probability function of the form

$$f_{\text{ZID}}(y)=\Pi_{0}\;I_{\left[y=0\right]}+(1-\Pi_{0})\;f_{D}(y;\theta )\;,$$

where *f*_*D*_(*y*|***θ***) is the probability function of distribution *D* with parameters ***θ***. Hence, *f*_ZID_(*y*) exhibits an additional, zero-inflation hyperparameter *Π*_0_for the proportion of additional zeros. From the equation above, the probability of zero is equal to *Π*_0_ + (1−*Π*_0_)*f*_*D*_(*y*=0|***θ***), while the probability of *y*>0 is given by (1−*Π*_0_)*f*_*D*_(*y*|***θ***).

Two popular models that account for data with excess zeros are the zero-inflated Poisson (ZIP) and the zero-inflated negative binomial (ZINB). The ZIP distribution introduced by Lambert [42] is the simplest ZID.

In the longitudinal setting, the full ZIP mixed effects model has the following representation:

$$\begin{array}{lll} Y_{\mathrm{ij}} & ∼\text{ZIP}(\Pi_{0\mathrm{ij}},\mu_{\mathrm{ij}})\;\text{and}\; & \;\\ Y_{\mathrm{ij}} & ∼\left\{\begin{array}{c} 0,\;\;\text{with probability}\;\;\Pi_{0,\mathrm{ij}}\\ \text{Poi}(\mu_{\mathrm{ij}}),\;\;\text{with probability}\;\;(1-\Pi_{0,\mathrm{ij}}). \end{array}\right)\; & \; \end{array}$$

A ZIP model will reflect the data accurately when overdispersion is caused by an excess of zeros. In general, a ZIP mixed effects model can be used when one is not certain about the nature of the source of zeros, and observations are overdispersed and simultaneously correlated because of the sampling design or the data collection procedure. By contrast, if overdispersion is attributed to factors beyond the inflation of zeros, a ZINB model is more appropriate [43]. Furthermore, the rate of zero-inflation may change over time. This problem goes beyond the scope of this paper, and we focus on ZIP GLMMs as an alternative to the Poisson GLMM generally used for analyzing longitudinal counts. More details concerning these issues can be found in Hilbe [44] or Lambert [42].

Again, a Bayesian approach provides an easy way to deal with zero-inflated hierarchical count data by incorporating a prior for *Π*_0_(generally beta prior or a uniform prior when no information is available). For longitudinal data with repeated observations, the correlation structure may be taken into account by introducing random effects in the proposed zero-inflated model. More details can be found in Dagne [45] or Ghosh *et al.*[46].

#### NMM with variance-stabilizing transformation

It is not uncommon for a regression model to be inappropriate for a given response variable but reasonable for some transformation provided that it is methodologically justified. For a longitudinal count outcome, this means that a *normal* mixed effects model (NMM) should be considered as an alternative modeling strategy, with an assumption of Gaussian errors on the transformed scale: an inverse hyperbolic sine-transformation [47] of the response *y* via

$$\text{arcsinh}(y)=\log(y+\sqrt{y^{2}+1})$$

can be performed to accomplish stabilization of the variance and is often useful in practice. For more details concerning the asymptotic variance-stabilizing transformation resulting from negative binomial count data, see the Appendix A1. This approach is motivated by analyzing the data with a robust and well-understood algorithm as regards parameter estimation. Particularly in a frequentist framework, likelihood-based inference is far less straightforward for GLMMs than it is for NMMs. Analytical intractability is the reason why a variety of numerical integration techniques for maximizing the likelihood have been developed (e.g. Gauss quadrature or penalized quasi-likelihood). In a Bayesian framework, computation is a major issue for complex hierarchical GLMMs since the usual implementation based on the Markov chain Monte Carlo (MCMC) method tends to exhibit poor performance, lack of convergence or slow mixing properties when applied for such models. As regards computational cost, NMMs clearly outperform mixed models for non-Gaussian response.

### Bayesian inference using the INLA approach

For Bayesian GLMMs, an analytical computation of the posterior marginals of the unknown fixed parameters and hyperparameters is not possible: The posterior marginals are not available in closed form because of the non-Gaussian outcome. Hence, the standard approach used to obtain posterior estimates are MCMC methods [48-50]. However, within the MCMC framework several problems in terms of both convergence and computational time occur in practical applications. Recently, Rue *et al.*[37] proposed an approximate alternative for parameter estimation in a subclass of Bayesian hierarchical models, the so-called *latent Gaussian models*. These are models with a structured additive predictor

(2) $$\eta_{i}=\alpha +\overset{n_{f}}{\underset{l=1}{\sum }}f^{\left(l\right)}(u_{\mathrm{li}})+\overset{n_{\beta }}{\underset{g=1}{\sum }}\beta_{g}x_{\mathrm{gi}}+\varepsilon_{i},$$

where *f*^(*l*)^(·) represents an unknown function of continuous covariates ***u***, comprising for example nonlinear effects of covariates, time trends, spatial dependencies, or independent identically distributed individual-level parameters (random effects). The *β*_*g*_’s denote the linear effect of some covariates ***x***, and the *ε*_*i*_’s are unstructured terms. Gaussian priors are assigned to *α*, *f*^(*l*)^(·), *β*_*g*_ and ***ε***, whereas the priors for the hyperparameters ***ϕ***do not have to be Gaussian. Random effects are introduced by defining *f*(*u*_*i*_) = *f*_*i*_ and letting {*f*_*i*_} be independent, have zero mean and be Gaussian distributed. INLA is a new computational approach to statistical inference for latent Gaussian Markov random field (GMRF) models that can bypass MCMC. Known problems with MCMC no longer apply using INLA as no Monte Carlo inference is involved. The theoretical background and computational issues are described in detail in Rue *et al.*[37,51]. In short, a latent GMRF model, which underlies INLA, is a hierarchical model which can be characterized through three stages. In the first stage, the distributional assumption is formulated for the observables *y*_*i*_, usually assumed to be conditionally independent given some latent parameters and, possibly, some additional hyperparameters. In the second stage, an a priori model for the unknown parameters is assigned and the corresponding GMRF is specified. The third and last stage of the model consists of determining the prior distributions for the hyperparameters. With this method, a recipe for fast Bayesian inference using accurate, deterministic approximations to the marginal posterior density for the hyperparameters and the marginal posterior densities for the latent variables is provided in a fully automated way. The INLA computational approach combines Laplace approximations and numerical integration in a very efficient manner. Three types of approximation are available: Gaussian, full Laplace, or simplified Laplace approximation. Each of these approaches has different features varying in accuracy and computational cost. In this article, we used the full Laplace approximation for the numerically inaccessible integrals of the posterior marginal density as this approximation is supposed to be the most accurate [37,52].

Using the INLA approach it is also possible to challenge the model itself. For example, a set of competing GLMMs can be assessed through cross-validation in a reasonable time without reanalyzing the model after omission of observation *y*_*ij*_. Hence, within the INLA framework, GLMMs can be fitted at low computational cost, giving access to various predictive measures for model comparison. Additionally, this approach facilitates the validation of distributional assumptions concerning the model being studied.

Details on how to use the open-source software inla can be found in the manual offered by Martino and Rue [38] or [53], and on the website http://www.r-inla.org. The inla-program, written in C and bundled within an R-interface [39] called R‐INLA, can be downloaded from the webpage for Windows, MAC and Linux, or simply by typing the following command line within Rsource("http://www.math.ntnu.no/inla/givemeINLA.R"). Accordingly, R‐INLA permits model specification and post-processing of results directly in R. All analyses in this paper were run using the R‐INLA package built in October 2011.

### Methods for model assessment and comparison

Diagnostic checking of the model against the data completes the model building process. The aim of diagnostic checking is to compare the data with the fitted model in such a way that it is possible to detect any systematic discrepancies. Forms of model assessment common in both frequentist and Bayesian methods involve measuring the goodness-of-fit to evaluate whether the chosen final model provides an adequate fit to the longitudinal data and to firmly establish the model’s credibility (*model assessment*). For example, we can check whether a specific random effect at a certain grouping level is warranted or whether it can be eliminated. To identify model deficiencies and facilitate model comparison and model selection, several Bayesian tools recently proposed by various authors are available. These tools can be applied to addressing the issue of predictive performance of a model, or to identify model deficiencies and to detect possible outliers or surprising observations *y*_*ij*_ that do not fit the given model and may require further attention. Additionally, methods for *model comparison* should provide information about which model performs best.

#### Deviance information criterion (DIC)

Appropriate statistical selection of the best model from a collection of hierarchical GLMMs is problematic mainly because of ambiguity in the “size” of such models arising from the shrinking of their random effects towards a common value. To address this problem, Spiegelhalter *et al.*[30] suggest DIC as a generalization of the Akaike information criterion (AIC) which can be used as a Bayesian approach for model comparison and to assess the adequacy of hierarchical models. DIC compares the global performance and predictive accuracy of different alternative models accounting for model complexity. Like AIC, the basic idea of DIC is a trade-off between model fit and model complexity. Models with more parameters tend to fit the data better than models with less parameters. However, a larger set of parameters makes the model more complex with the danger of overfitting. Hence, model selection should account for both goodness-of-fit and complexity of the model. The smaller the DIC the better the trade-off between model fit and complexity. The model with the smallest DIC is considered to be the model that would best predict a replicate data set of the same structure as that currently observed. DIC is based on the posterior distribution of the *Bayesian deviance* statistic,

(3) D(θ)=−2logf(y|θ)+2logh(y),

where *f*(*y*|*θ*) is the likelihood function for the observed data vector *y* given the parameter vector *θ*, and *h*(*y*) is some standardizing function of the data (thus not having an impact on model selection). In this approach, the fit of a model is summarized by the posterior expectation of the deviance $\left(\bar{D}={E\;}_{\theta |y}[D]\right)$, while the complexity of a model is captured by the effective number of free parameters *p*_*D*_, which is typically less than the total number of parameters. For non-hierarchical models, *p*_*D*_ should be approximately the true number of parameters. *p*_*D*_can be thought of as the *“posteriori mean of the deviance”* minus the *“deviance evaluated at the posterior means”*

$$p_{D}={E\;}_{\theta |y}[D]-D({E\;}_{\theta |y}[\theta ])=\overset{Â¯}{D(\theta )}-D(\bar{\theta }).$$

DIC is then defined as

(4) $$\begin{array}{lll} \text{DIC} & =2\;\bar{D}-D(\bar{\theta })=D(\bar{\theta })+2\;p_{D}\; & \;\\ & =\bar{D}+p_{D},\; \end{array}$$

DIC is scale-free. Because of the standardizing function *h*(*y*) in (3), DIC values have no intrinsic meaning, and only differences in DIC across candidate models are meaningful. The question of what constitutes a noteworthy difference in DIC between two candidate models has not yet received a satisfactory answer. Differences of 3 to 5 are normally being thought of as the smallest that are still noteworthy [49,50].

Spiegelhalter *et al.*[30] and Plummer [54] discuss some limitations of DIC: Although widely used, DIC lacks a clear theoretical foundation. It can be shown that DIC is an approximation of a penalized loss function based on the deviance, with a penalty from a cross-validation argument. However, this approximation is valid only when the effective number of parameters in the model is much smaller than the number of independent observations. The ratio *p*_*d*_/*n*may be used as an indicator of the validity of DIC. In disease mapping or random effects models for longitudinal data this assumption often does not hold and therefore DIC under-penalizes more complex models.

##### Computational details

DIC is simple to calculate using MCMC simulation and is routinely implemented in WinBUGS[55-58]. With the INLA approach, both components of DIC, *p*_*D*_and $\left(\bar{D}\right)$, can be computed by setting the option dic = TRUE in the control.compute statement within the inla(.)-call. For further details see [37].

#### Conditional predictive ordinate (CPO)

As a device for detecting possible outliers or surprising observations *y*_*ij*_ within a posited model and therefore checking the model fit, the *conditional predictive ordinate* (CPO) for each observation can be computed [59]. To be more precise, this predictive quantity given by

(5) $$\mathrm{CP}O_{\mathrm{ij}}=\Pi (y_{\mathrm{ij}}|y_{-\mathrm{ij}})$$

constitutes the position of the observed value *y*_*ij*_within the leave-one-out cross-validatory posterior predictive distribution evaluated at the observed value *y*_*ij*_. A small value of *CPO*_*ij*_ indicates an observation *y*_*ij*_that is unlikely under the model fit without the observation in question, i.e. ’surprising’ in the light of the prior knowledge and the other observations [60]. Accordingly, this observation is not expected under the cross-validation posterior predictive distribution of the current model. CPO measure is discussed among others by Gelfand *et al.*[32], Congdon [61] and Gilks *et al.*[48]. Since for discrete data *CPO*_*ij*_ can be used to estimate the probability of observing *y*_*ij*_in the future when ***y***_−*ij*_is already observed, it can be interpreted easily.

##### Computational details

Examination of model performance at the level of the individual observation can provide added insight into discovering the reasons for poor global performance. For each observation *y*_*ij*_ of the model, we use the value of the cross-validation predictive density at the observed data points as a local discrepancy measure. A plot for *CPO*_*ij*_versus *ij* can be used informally as a diagnostic tool to reveal surprising observations. With MCMC sampling, calculating the CPO predictive quantity requires refitting the model by single case omission. With inla, CPO can be returned for each observation at low computational cost without rerunning the analysis by using the option cpo = TRUE. However, in practice, the assumptions behind the numerical computation might fail for some observations. For these points, the CPO values have to be re-computed manually. That is, *y*_*ij*_ is removed from the model and simply refitted only computing the posterior marginals for the linear predictor for this observation. (As the results from fitting the whole model can be used to improve e.g. initial values, this process allows a more efficient implementation). For further reading refer to [51].

#### Proper scoring rules as a toolbox for the assessment of prognostic performance

Besides model choice criteria such as DIC, CPO or graphical techniques, the comparison and ranking of different competing models can be based on *proper scoring rules* which were proposed by Gneiting and Raftery [33] for assessing the quality of probabilistic forecasts (see [34-36,62] for more details). Scoring rules provide a suitable summary measure for the evaluation of probabilistic forecasts, by assigning a numerical score based on the posterior predictive distribution *P* and on the event *y* that materializes. We take scoring rules to be negatively oriented penalties that a forecaster wishes to minimize: Specifically, if the forecaster quotes the predictive distribution *P* and *y* is the observed value, the penalty is *s*(*Py*). We write *s*(*PQ*) for the expected value of *s*(*PY*), when *Y*∼*Q*. Models with smaller score values should be preferred to models with larger values. Additionally, propriety is an essential property of a scoring rule that encourages honest and coherent predictions. Gneiting and Raftery [33] contend that the goal of probabilistic forecasting is to maximize the sharpness of the predictive distributions subject to calibration. *Calibration* refers to the statistical consistency between the probabilistic forecasts and the observations *y*, and is a joint property of the predictive distributions and the actual observation *y*. *Sharpness* refers to the concentration of the predictive distribution, and is a property of the forecast only [28,29,63]. Hence, in the context of model comparison, scoring rules provide a diagnostic approach to assessing the predictive performance of a model.

The most prominent example of strictly proper scoring rules is the *logarithmic score*[64] which is defined as

(6) $$LS_{\mathrm{ij}}=-\log(\Pi_{y_{\mathrm{ij}}})\;,$$

where $\left(\Pi_{y_{\mathrm{ij}}}=\text{Pr}(Y_{\mathrm{ij}}\;\;=\;\;y_{\mathrm{ij}}\;|\;y_{-\mathrm{ij}})\right)$ indicates the cross-validated leave-one-out predictive probability mass at the observed value *y*_*ij*_, *i*=1,…,*N*, *j*=1,…,*n*_*i*_. The subscript −*ij* in ***y***_−*ij*_ denotes that for patient *i* observation *j* is removed. Concerning logarithmic score values, the following relationship holds:

$$LS_{\mathrm{ij}}=-\log(\mathrm{CP}O_{\mathrm{ij}}).$$

Gneiting and Raftery [33] proposed ranking competing forecast procedures (i.e. competing models) on the basis of their mean scores, e.g. $\left(\overset{Â¯}{\mathrm{LS}}={\left(\overset{N}{\underset{i=1}{\sum }}n_{i}\right)}^{-1}\underset{i,j}{\sum }LS_{\mathrm{ij}}\right)$, and not by graphical methods such as boxplots. The difference in the mean scores can be considered since only the mean scores are still proper. Therefore, we want to compare the mean scores of two rival models by using a formal significance test to assess if score differences are statistically significant on a certain level. The paired Monte Carlo permutation test [65,66] based on the observation-level scores provides a convenient approach, as unlike the paired *t*-test it does not require distributional assumptions (e.g. normality of individual scores) or trust in asymptotic behavior. Permutation tests compare the observed score values, suitably summarized in a test statistic, with randomly permutated score values, which can be viewed as samples under the null hypothesis *H*_0_ of no difference.

##### Computational details

To calculate the scores in the MCMC setting, a leave-one-out cross-validation approach using the posterior predictive distribution is the gold standard, obtained by reanalyzing the data without a suspect statistical unit. However, full and exact cross-validation is extremely time-consuming in practice and often generally infeasible within an MCMC analysis. Marshall and Spiegelhalter [67] proposed the “full-data mixed approach” (ghost sampling) generating full ’ghost’ sets of random effects for each unit without repeatedly fitting the model with one particular observation removed (for more details also compare [68] or [69]).

As alternative to MCMC, the INLA approach can be applied to compute omnibus predictive performance measures such as the mean cross-validated logarithmic score of different competing models. Using inla, the quantities needed for calculating these score values are available as by-product of the main computations when setting the option cpo = TRUE in the control.compute statement within the inla(.)-call [37,38,51].

#### Probability integral transform (PIT)

Unusually small values of CPO indicate surprising observations. However, what is meant by ’small’ has to be calibrated to the level of the Gaussian field in order to compare CPO values. One possible calibration procedure is to compute the *probability integral transform* (PIT) proposed by Dawid [27]. In the univariate case, PIT is a tool for assessing calibration and therefore evaluates the adequacy of a single model.

The PIT value for each single observation is defined as

(7) $${\text{PIT}}_{\mathrm{ij}}=\text{Pr}\;(y_{\mathrm{ij}}^{\text{new}}\leq y_{\mathrm{ij}}|y_{-\mathrm{ij}})\;,$$

with ***y***_−*ij*_being the observation vector with the *ij*th component omitted, and is simply the value that the predictive cumulative distribution function (CDF) attains at the observation *y*_*ij*_. This procedure is performed in cross-validation mode meaning that in each step of the validation process the ensuing leave-one-out posterior predictive distribution is calculated.

Unusually large or small values of PIT_*ij*_indicate possible outliers or surprising observations not supported by the model under consideration. If the observation is drawn from the predictive distribution, which is an ideal and desirable situation, and the predictive distribution is continuous, the PIT has a uniform distribution on the unit interval [0,1]. To evaluate whether a data vector ***y***seems to come from a specific distribution, i.e. to check calibration empirically, a histogram of all PIT values can be plotted and checked for uniformity [28,29,33]. A histogram of the PITs far from the uniform might indicate a questionable model and hint at reasons for forecast failures and model deficiencies. U-shaped histograms indicate under-dispersed predictive distributions, inverse-U shaped histograms point at over-dispersion, and skewed histograms occur when central tendencies are biased. In the case of count data, the predictive distribution is discrete resulting in PIT values no longer being uniform under the null hypothesis of an ideal forecast. To overcome this problem, several authors suggest a “non-randomized” version of PIT values (see [28] for more technical details). Hence, an *adjusted* PIT can be used instead, defined as

(8) $${\text{PIT}}_{\mathrm{ij}}=\mathrm{Pr}\;(y_{\mathrm{ij}}^{\text{new}}<y_{\mathrm{ij}}|y_{-\mathrm{ij}})+0.5\cdot \mathrm{Pr}\;(y_{\mathrm{ij}}^{\text{new}}=y_{\mathrm{ij}}|y_{-\mathrm{ij}})\;,$$

These adjusted PIT values can be interpreted in exactly the same way as in applications with continuous outcome data. However, when using PIT as a diagnostic tool it has to be considered that PIT does not take into account the sharpness of the density forecast, as opposed to proper scoring rules providing a combined assessment of both calibration and sharpness simultaneously.

##### Computational details

In the MCMC setting, nonrandomized PIT values for count outcomes are cumbersome and rather time consuming because of the leave-one out cross-validation approach. To reduce the computational burden, the INLA approach can be applied to compute PIT_*ij*_, *i* = 1,…,*N*, *j* = 1,…,*n*_*i*_.

#### Motivating example: vertigo phase III dose-finding study (BEMED trial)

##### Study synopsis

The BEMED trial (Medical treatment of Menière’s disease with Betahistine; EudraCT No.: 2005-000752-32; BMBF177zfyGT; Trial Registration: Current Controlled Trials ISRCTN44359668) is an ongoing investigator-initiated, multi-center, national, randomized, double-blind, placebo-controlled, clinical trial with a parallel group design. This dose-finding phase III trial recruiting patients from several dizziness units throughout Germany comprises three arms: therapy with high dose betahistine-dihydrochloride (3×48 mg per day) vs. a low dose (2×24 mg per day) vs. placebo. Total treatment time will be nine months with a three month follow-up. The objective of this study is to evaluate the effects of betahistine in high-dosage vs. low-dosage vs. placebo on the occurrence of vertigo attacks. The study was approved by the local ethics committee and is performed in accordance with the International Conference on Harmonization Guidelines for Good Clinical Practice, as well as with the Declaration of Helsinki. Written informed consent was obtained from patients who met the study inclusion criteria.

##### Design aspects and statistical analyses

A sample size of *n* = 138 patients in total (46 in each group) to be analyzed was considered necessary. The total treatment time will be nine months with a three month follow-up. The primary efficacy outcome is the number of vertigo attacks in the three treatment arms during the last three months of the 9 month treatment period. The primary efficacy analysis is nonparametric and will be performed according to the ITT principle. The closed testing procedure is used to avoid adjusting the significance level. Sensitivity analyses will be performed using a longitudinal approach to quantify patient profiles and the ’speed of efficacy’, i.e., how quickly reduction in attack frequency is achieved in the three treatment groups. For the prospectively specified SAP, it has to be decided which candidate set of mixed effects models proposed at the beginning of the Methods Section seems appropriate for analyzing the counts.

##### Informed model choice

The decision on the models for sensitivity and ancillary analyses and handling of informative missing data of the large phase III BEMED trial is based on data from a *pre-study* with a comparable study population, comparable intervention, and same definition of primary outcome (frequency of vertigo attacks).

## Results

### Application to clinical trial data

#### Vertigo pre-study: data and modeling details

**Figure 1:**
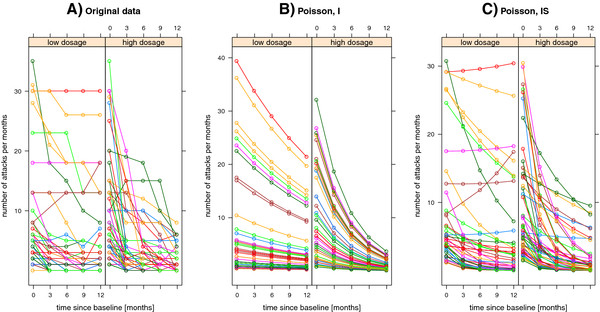
**Trajectory plots for vertigo data.** Effect of betahistine-dihydrochloride on the frequency of attacks of vertigo in a total of 112 Menière’s disease patients; 2 treatment groups: “low-dosage” (50 patients) vs. “high-dosage” (62 patients). **A)** individual trajectories for vertigo data. **B)** and **C)** display the conditional posterior mean trajectories of the number of attacks depending upon fixed and random effects after fitting a Poisson GLMM (I: model with random intercepts. IS: model with random intercepts and slopes). The same color is used to indicate observations and model-based estimates for the same patient.

To demonstrate the applicability of the Bayesian toolbox within the GLMM framework, we used real life longitudinal count data from an open, non-masked, *exploratory* trial conducted by the dizziness unit, Department of Neurology, University Hospital Munich, Germany [70]. 112 patients between the ages of 18 and 80 years with Menière’s disease received either a low dosage of betahistine-dihydrochloride, i.e. 16 or 24 mg tid, or a higher dosage of 48 mg tid for at least 12 months. 50 patients were in the low dosage group (coded as zero) and 62 in the high dosage group (coded as one). Both treatment groups did not differ with respect to patient characteristics at baseline measurement (*t*_*i*1_≡*t*_1_=0, for all *i* = 1,…,112). In particular, there was no significant difference in the number of attacks at baseline (see [70] for more details). The full dosage was given from the beginning of the treatment. Since the major aim of the treatment of Menière’s disease is reducing the attack frequency, the efficacy outcome variable was the number of vertigo attacks per month during a 3-month period, i.e. during a period of 3 months preceding treatment and then every 3 months for up to 12 months. Follow-up examination every 3 months showed that the mean number of attacks per month decreased in both groups over time, and was significantly lower in the high-dosage than in the low dosage group; the longer the treatment, the greater the difference between the two treatment groups. Longitudinal data are displayed in Figure 1. Moderate vertical differences between the individual profiles could be identified.

We consider a count outcome variable, *y*_*ij*_, which in our example represents the number of vertigo attacks per months for the *i*th patient measured at time *t*_*ij*_≡*t*_*j*_ = 0,3,6,9,12, for *j* = 1,2,3,4,5. To account for between-patient variability we introduced patient-level random intercepts as well as patient-specific slopes, and then fitted main effects and interaction models:

$$\begin{array}{lll} \eta_{\mathrm{ij}} & =(\beta_{0}+b_{0i})+\beta_{1}\;{\text{time}}_{j}+\beta_{2}\;{\text{dosage}}_{i}\cdot {\text{time}}_{j}\;\;\;\;\text{model (I)}\; & \;\\ \eta_{\mathrm{ij}} & =(\beta_{0}+b_{0i})+(\beta_{1}+b_{1i})\;{\text{time}}_{j}+\beta_{2}\;{\text{dosage}}_{i}\cdot {\text{time}}_{j}\;\text{model (IS)}\; & \; \end{array}$$

The main effect for a treatment group, defined by dosage_*i*_, was left out of the systematic part since treatment effect was expected to happen slowly with time and not in a way that a strong effect is established after a short time and stays stable for the duration of the longitudinal observation.

We considered flexible models allowing for overdispersion and zero-inflation, respectively. Hence, both for models of type (I) and (IS), we investigated four different types of GLMM by changing the distributional assumption: 

a) *Poisson* model for *y*_*ij*_∼Poi(*μ*_*ij*_). Poisson GLMM was used as the “reference model” as this distributional assumption is often the default choice.

b) *Zero-inflated Poisson* (ZIP) model, which will explain the mean attack frequency and the zero-inflation probability (i.e. assuming an excess of zero observations).

c) *Negative Binomial* (NB) model, as a robust alternative to accommodate substantial extra variation or overdispersion.

d) *Normal* mixed effects model (NMM), for arcsinh-transformed outcome “attack frequency” as an alternative modeling strategy to accomplish stabilization of variance.

All models included patient-specific random intercepts $\left(b_{0i}|\sigma_{b}^{-2}{∼}_{\mathrm{iid}}N(0,\sigma_{b}^{2})\right)$, while the need for patient-specific slopes associated with time was investigated for all candidate models. Therefore, for the latter type of GLMM, correlated patient-specific intercepts and slopes being zero mean bivariate normal were assumed, i.e. $\left({\left(b_{0i},b_{1i}\right)}^{T}|Q{∼}_{\mathrm{iid}}N_{2}(0,Q^{-1})\right)$. For models of type (I), a Gamma prior was assigned to the precision $\left(\sigma_{b}^{-2}\right)$. According to Fong *et al.*[40], for models of type (IS) we assumed **Q**to follow a $\left({\text{Wishart}}_{2}(r,R^{-1}\right)$)-distribution with **Q** = **I**_2_. In general, independent zero-mean Gaussian priors with fixed small precisions were assigned to each component of the population-level parameter vector ***β***. As the accuracy of the simplified Laplace approximation is often not sufficient for the computation of predictive measures [52], the full Laplace approximation was chosen in the following application, in combination with the so-called GRID integration strategy for numerically exploring the approximative posterior marginal densities (for more details concerning this issue see [37]).

#### Vertigo pre-study: analysis results

In Table 1 INLA summaries for the vector of population-level parameters (fixed effects) are described. Additionally, 95% credible intervals are reported. These 95% equal-tail intervals correspond to the 2.5% and 97.5% percentiles of the corresponding posterior distribution and enable assessment of whether, e.g., time profiles of the primary efficacy outcome variable differ in both treatment groups (dosage⋆time). We conclude that posterior estimates for models of type (I) and of type (IS), respectively, agree between differing distributional assumptions.

**Table 1:** INLA summaries for estimated posterior means of population-level parameters (together with 2.5% and 97.5% posterior quantiles) using full Laplace approximations

	**Parameter**		
**Model**	**Intercept**	**time**	**dosage⋆time**
Poisson, I	1.366 (1.123, 1.603)	-0.051 (-0.063, -0.038)	-0.130 (-0.150, -0.109)
Poisson, IS	1.638 (1.432, 1.837)	-0.189 (-0.275, -0.107)	-0.173 (-0.288, -0.061)
ZIP, I	1.375 (1.121, 1.620)	-0.049 (-0.062, -0.036)	-0.115 (-0.137, -0.093)
ZIP, IS	1.628 (1.421, 1.830)	-0.209 (-0.302, -0.119)	-0.198 (-0.323, -0.075)
NB, I	1.447 (1.193, 1.695)	-0.069 (-0.090, -0.050)	-0.127 (-0.156, -0.098)
NB, IS	1.642 (1.433, 1.840)	-0.190 (-0.289, -0.101)	-0.168 (-0.289, -0.049)
arcsinh^∗^, I	2.056 (1.853, 2.259)	-0.067 (-0.084, -0.051)	-0.074 (-0.096, -0.052)
arcsinh^∗^, IS	2.055 (1.854, 2.255)	-0.068 (-0.114, -0.022)	-0.073 (-0.134, -0.012)

∗ INLA posterior means and 95% credibility intervals for arcsinh-transformed outcome modeled as continuous response with Gaussian error terms.

I: model with random intercept, IS: model with random intercept and slope associated with time.

**Figure 2:**
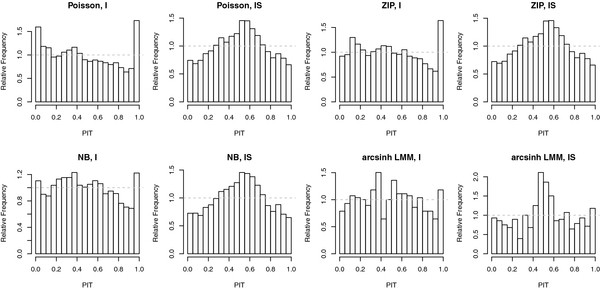
**Vertigo data: INLA vs. MCMC approach.** Bayesian inference for fixed effects (Poisson random slope model): comparison of samples from a long MCMC chain (□) with the posterior marginals computed with the Laplace approximation (—) obtained by using INLA. The vertical blue line shows the posterior mean.

In Figure 1B) and C), the conditional mean estimates of the number of attacks depending upon fixed effects for time, interaction for treatment group and time, and random effects are visualized by means of trajectory plots, assuming a Poisson (IS) GLMM. Furthermore, Figure 2 illustrates the approximated posterior marginals for the most important fixed effects by comparing INLA results with those obtained using the MCMC approach (see Appendix A3).

**Figure 3:**
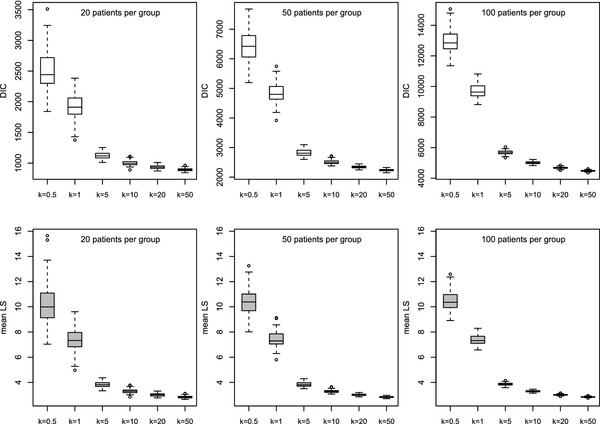
**Vertigo data: PIT histograms for all candidate models.** U-shaped histograms indicate under-dispersed predictive distributions, hump or inverse-U shaped histograms point at overdispersion, and skewed histograms occur when central tendencies are biased. Dashed gray lines show the histogram height corresponding to perfect calibration.

However, our key scientific problem was to quantify the goodness of competing models in terms of prediction accuracy. The question to be answered was how structural differences concerning random effects or distributional assumptions affect the performance of a posited model. Calibration check was performed by PIT histograms serving as an informal tool for discordancy diagnostics (see Figure 3). In contrast to NB GLMM and arcsinh NMM, which seem to be sufficiently well calibrated for type (I)-models, the Poisson (I) and the ZIP (I) model were slightly U-shaped, indicating a worse predictive performance for higher columns at the right-hand end of the histograms. Visual assessment of PIT histograms for type (IS)-models revealed noticeable deviations from uniformity due to miscalibration of density forecasts.

Additionally, competing random slope models did not clearly outperform each other. The difference between negative binomial and Poisson was marginal because of a small degree of overdispersion: e.g. the hyperparameter *k* was estimated to be rather large for the NB (I) model with random intercepts, with a posterior mean of 7.03, 95% credible interval [4.65,10.36]. For the NB (IS) model, the posterior estimates for *k* were even larger (data not shown). Likewise, there was no convincing evidence for zero-inflation (e.g. the posterior mean for zero-probability hyperparameter *Π*_0_ was estimated to be 0.09, 95% credible interval [0.05, 0.14], for ZIP (I). For model type ZIP (IS), the posterior mean for *Π*_0_ was even less). Modeling the arcsinh-transformed outcome by means of an NMM has several computational advantages, and the PIT values seemed reasonably close to uniform for model type (I), hence yielding fairly correct forecasts. Nevertheless, this does not take the sharpness of the density forecasts into account, as opposed to proper scoring rules.

Table 2 enables a comparison of $\left(\overset{Â¯}{\mathrm{LS}}\right)$ and DIC for all eight types of GLMM (the lowest mean score and DIC is printed in bold face). The Poisson (IS) model was ranked best in the leave-one-out predictive assessment by the logarithmic score. A permutation test was applied to decide whether the difference in mean log scores was significant on a 5% level. More exactly, we used the Poisson (IS) model emerging with the lowest $\left(\overset{Â¯}{\mathrm{LS}}\right)$ as the reference model and tested in a pairwise manner. The last column of Table 2 depicts Monte Carlo *p*-values based on permutation tests (9999 permutations) for comparison of $\left(\overset{Â¯}{\mathrm{LS}}\right)$ for the Poisson (IS) GLMM with $\left(\overset{Â¯}{\mathrm{LS}}\right)$ if the remaining competing models are chosen for data analysis. n.a. means that a permutation test is not applicable because of backtransformation of $\left(\overset{Â¯}{\mathrm{LS}}\right)$ obtained within the arcsinh-NMM (see Appendix A2. Backcalculation of the mean logarithmic score for arcsinh-transformed outcome to the original count scale for further details).

**Table 2:** **Posterior mean of the deviance (**$\left(\bar{D}\right)$**), deviance of the mean (**$\left(D(\bar{\theta })\right)$**), effective number of parameters (***p*_*D*_**) as measure of model complexity, DIC value, and mean of logarithmic scores (**$\left(\overset{Â¯}{\text{LS}}\right)$**)**

**Model**	$\left(\bar{D}\right)$	$\left(D(\bar{\theta })\right)$	***p***_***D***_	**DIC**	$\left(\overset{Â¯}{\mathrm{LS}}\right)$	***p*-value**^***†***^
Poisson, I	2034	1931	103	2137	2.046	<0.0001
Poisson, IS	1633	1463	170	**1803**	**1.641**	ref.
ZIP, I	2020	1917	103	2123	2.018	<0.0001
ZIP, IS	1637	1467	170	1807	1.644	0.5797
NB, I	2006	1903	103	2109	1.932	<0.0001
NB, IS	1659	1489	170	1829	1.665	0.6841
arcsinh, I	994^∗^	886^∗^	108^∗^	1102^∗^	1.967	n.a.
arcsinh, IS	834^∗^	636^∗^	198^∗^	1032^∗^	1.960	n.a.

∗ Comparison of DIC for NMMs is not applicable because of different arcsinh-transformed outcomes.

*†*Monte-Carlo permutation test for paired individual logarithmic scores.

The Poisson (IS) model and the negative binomial (IS) counterpart do not differ significantly with respect to their mean cross-validated logarithmic scores. The same holds for the ZIP (IS) alternative. Ranking these models by means of their DIC value (disregarding NMM types) revealed that they are very close to each other.

In summary, there was no evidence of considerable over-dispersion and excess of zeros. Inclusion of a zero-inflation component is apparently not necessary for these pre-study data. Applying mean log score and DIC to rank all eight models considered so far suggests that random intercept models are inferior to random intercept and slope models.

Hence, we are inclined to believe that a Poisson random intercept and slope model is suitable for these longitudinal count response data.

#### SAP for BEMED trial: selection of candidate models

After detailed analyses of the pre-study data described above, we will present these results in the SAP and choose a negative binomial model with random intercepts as well as random slopes as a robust candidate to conduct sensitivity analyses for the efficacy data of the BEMED trial. Accordingly, this proposed modeling strategy will be determined in the SAP.

### Simulation study

#### Sampling details

In the last section, a prediction-oriented Bayesian toolbox was applied to real-life clinical count data. It is also important to investigate whether these tools help to evaluate different model alternatives and whether the model comparisons are valid. To assess the discriminatory power as well as the properties of DIC and mean logarithmic score in the longitudinal count response situation, a simulation study was carried out. Following the real data structure of our clinical trial about patients with vertigo attacks, a parallel group design was assumed with four measurements occurring at times *t*=(*t*_1_,*t*_2_,*t*_3_,*t*_4_)=0,1,2,3 (exactly balanced design) for all subjects. There are two groups each of size *n*, with different fixed time slopes, parameterized by *β*_1_=−0.3 and *β*_2_=−0.5, but equal starting points at time *t*_1_=0. To be more detailed, we considered repeated count outcomes to follow a negative binomial distribution, conditioned on the random effects. Accordingly, the true sampling model is *Y*_*ij*_|*μ*_*i*_∼_*iid*_NB(*k*,*p*_*i*_), *i*=1,…,2*n*, *j*=1,…,4. To account for patient-specific variability, a random intercept *a*_*i*_was introduced, so the model can be summarized as

$$\log\mu_{\mathrm{ij}}=\alpha +a_{i}+t_{\mathrm{ij}}[\;\beta_{2}G_{i}+\beta_{1}(1-G_{i})\;],$$

with $\left(a_{i}|\sigma_{a}^{-2}∼N(0,\sigma_{a}^{2})\right)$, and *G*_*i*_representing the placebo and the verum group, respectively. The standard deviation of the random intercept was set to *σ*_*a*_ = 0.3 and the population intercept fixed at *α* = 3. The following candidate GLMMs are ranked by DIC as well as evaluated with respect to their forecasting capability: 

● negative binomial (true data generating distribution),

● Poisson,

● zero-inflated Poisson,

● zero-inflated negative binomial,

● NMM for arcsinh-transformed count outcome.

The ZIP and ZINB model were chosen to investigate whether the zero-inflated component improves the model performance. To define simulation scenarios we varied the sampling size (*n*, numbers per group) and the degree of over-dispersion as follows: *n* = 20,50,100 and *k* = 0.5,1,5,10,20,50. By combining the possible values of the sample size and the overdispersion parameter *k*, we therefore obtain count data for 18 different simulation scenarios which can be analyzed for all five rival models described above. Each model scenario provided *r* = 100 simulation runs to assess the variability of results.

All analyses were performed using the INLA approach. To get reliable results and to enhance the accuracy of Bayesian predictive measures (i.e. DIC and logarithmic CPO values), the full Laplace approximation in combination with the so-called GRID integration scheme was chosen as the strategy for deterministic approximation of the latent Gaussian field and the posterior marginals of the hyperparameters. For more details of estimation procedure, the reader is referred to [37,51] and the Additional files 1 and 2 (Supplementary Material).

When working with small data sets, the prior distribution can become influential in the posterior results, especially with respect to the spread of the posterior distribution, even if non-informative settings are chosen. This can particularly be an issue with prior distributions on the variance components. Therefore, for prior specification we followed the procedure outlined in [40] so as not to favor one modeling strategy over another. We assumed a marginal Cauchy distribution for the patient-specific intercept *a*_*i*_. A 95% range of [−0.6,0.6] for *a*_*i*_ gives a prior $\left(\sigma_{a}^{-2}∼\right)$ Ga(0.5, 0.001115), and hence, integration over $\left(\sigma_{a}^{-2}\right)$ gives the marginal distribution of *a*_*i*_as *t*_1_(0,0.00223,1).

#### Simulation results

**Figure 4:**
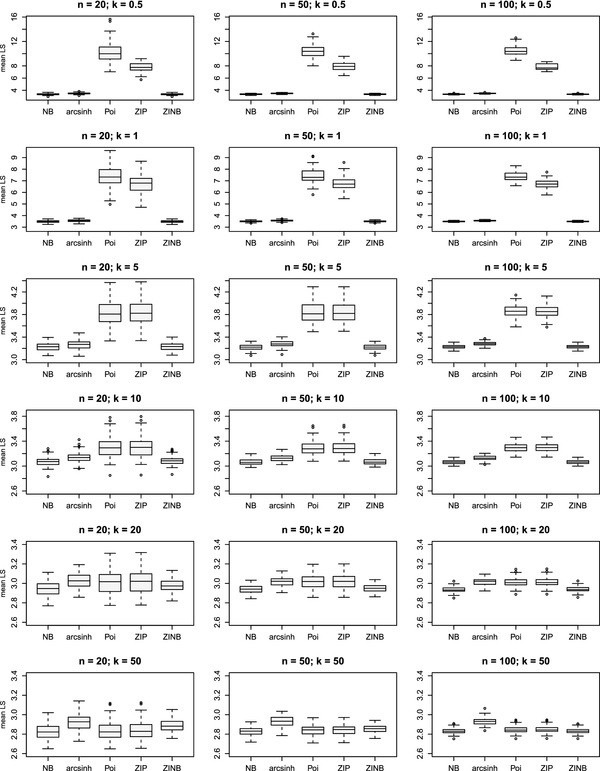
**Simulation study: Discriminatory power of DIC and **$\left(\overset{Â¯}{\text{LS}}\right)$**for different scenarios (100 runs per scenario).** Data generating process: longitudinal, negative binomial counts with subject-specific intercept (balanced design); modeling strategy: Poisson GLMM with random intercept; number of subjects per group: *n* = 20,50,100; degree of overdispersion: *k* = 0.5,1,5,10,20,50. As *k*→*∞*, the degree of overdispersion decreases and the negative binomial converges to a Poisson distribution. Hence, DIC and $\left(\overset{Â¯}{\mathrm{LS}}\right)$ decline. Note that the range of DIC increases in the case of a larger sample size.

Figure 4 depicts boxplots for different simulation scenarios if count response data $\left(\left\{y_{\mathrm{ij}}^{\left(r\right)}\right\}\right)$, *i*=1,…,2*n*; *j*=1,…,4;*r*=1,…,100, are analyzed by choosing a Poisson GLMM, i.e. a wrong modeling strategy in the case of high overdispersion. Both DIC as a measure of model selection and mean of logarithmic score ($\left({\overset{Â¯}{\mathrm{LS}}}^{\left(r\right)}\right)$, *r* = 1,…,100) were calculated for all 100 runs. The striking feature of these plots is that for all 6 setups, DIC and $\left(\overset{Â¯}{\mathrm{LS}}\right)$ discriminate strongly between the wrong model and the true negative binomial model generating the counts. If a Poisson model is chosen for data with a considerably high amount of overdispersion (small *k*), higher score values are assigned to the predictive distribution. DIC is clearly influenced by the sample size because of the deviance measure depending on the likelihood, whereas for $\left(\overset{Â¯}{\mathrm{LS}}\right)$ the number of sampling units does not impact scaling of the mean of the scores.

Based on these simulations, we conclude that DIC and $\left(\overset{Â¯}{\mathrm{LS}}\right)$ provide a suitable measure for ranking and evaluating model alternatives defined by different error distributions or variance structures.

**Figure 5:**
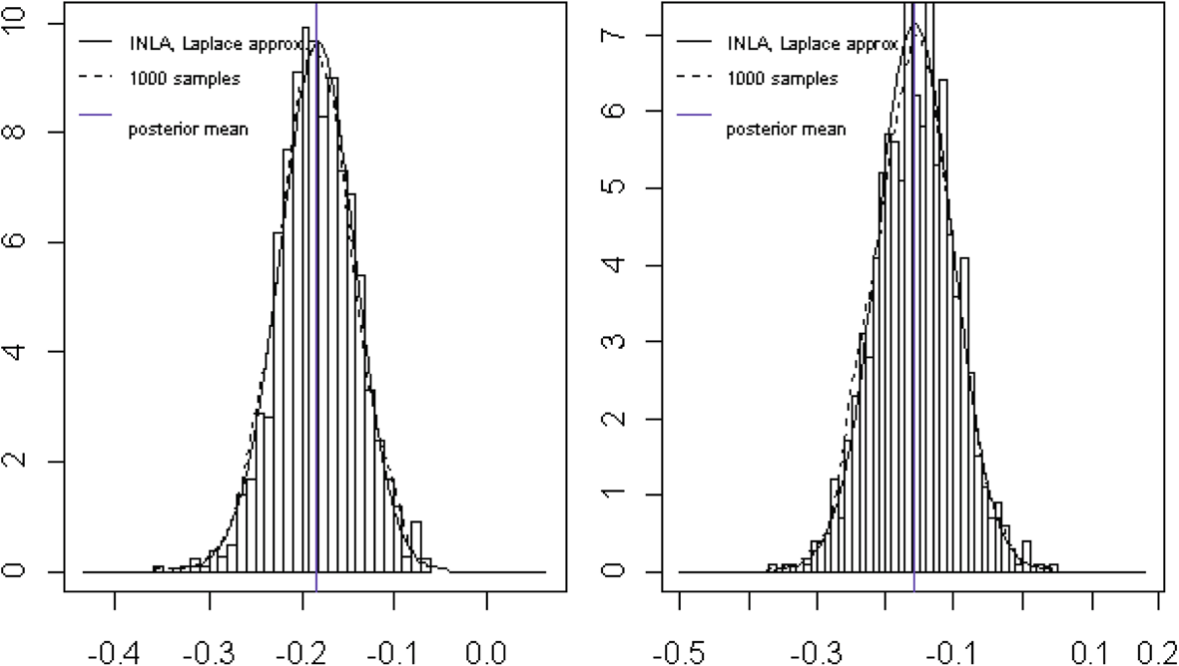
**Simulation study: Variability of mean LS within different simulation scenarios.** Variability of mean logarithmic score $\left({\overset{Â¯}{\mathrm{LS}}}^{\left(r\right)}\right)$ for true negative binomial (NB) compared with arcsinh, (zero-inflated) Poisson and zero-inflated NB model (*r* = 1,…,100 iterations per scenario). Sample size: *n* = 20,50,100 subjects per group; *k* = 0.5,1,5,10,20,50 determines the amount of overdispersion. Each row is a different value of *k* (amount of overdispersion), and each plot shows the mean $\left({\overset{Â¯}{\mathrm{LS}}}^{\left(r\right)}\right)$ for each competing model. The column of panels on the right has the largest sample size *n*; the top row exhibits the results for highly overdispersed counts (*k*=0.5).

For all three sample size situations (i.e. 40, 100, 200 units in total), Figure 5 reveals the difference in mean log scores for the true negative binomial GLMM compared with the following model alternatives: Poisson (neglecting over-dispersion), zero-inflation (assuming an excess of zeros) and a Gaussian response model after arcsinh-transformation of the counts *y*_*ij*_. The inadequacy of the Poisson model in terms of probabilistic forecasting is evident in the case of high overdispersion, denoted by the parameter *k*. If *k*→*∞*, $\left(\overset{Â¯}{\mathrm{LS}}\right)$ of the “wrong” Poisson model approximates the mean log score of the true NB model.

Furthermore, Table 3 reports the area underneath the receiver operating curve (AUC) as a summary measure for $\left({\overset{Â¯}{\mathrm{LS}}}^{\left(r\right)}\right)$, *r* = 1,…,100, of the true NB model and a competing model alternative, as displayed in Figure 5. For each combination of *k* (degree of overdispersion) and *n* (sample size) the discriminatory power of the mean log score was investigated. Perfect discrimination corresponds to an AUC value of 1 while random discrimination corresponds to an AUC value of 0.5. The AUC can be interpreted as being equal to the probability that $\left(\overset{Â¯}{\mathrm{LS}}\right)$ of the wrong model exceeds that of the true NB model, i.e. the probability that the wrong model has a lower predictive performance compared with the true data generating distribution. Accordingly, it is the probability that test results from a randomly selected pair of $\left({\overset{Â¯}{\mathrm{LS}}}^{\left(r\right)}\right)$ values for the wrong model and the true NB model are correctly ordered, namely $\left(\text{Pr}({\overset{Â¯}{\mathrm{LS}}}^{\left(r,\text{wrong}\right)}>{\overset{Â¯}{\mathrm{LS}}}^{\left(r^{\prime },\text{true}\right)}),r\neq r^{\prime }\right)$.

**Table 3:** **Area under the curve (AUC) for comparison of mean logarithmic score **$\left({\overset{Â¯}{\text{LS}}}^{\left(r\right)}\right)$**of true vs. wrong modeling strategy (*r* = 1,…,100 iterations per simulation scenario)**

***k*, degree of**		**AUC**		
**overdispersion**	**Model**	***n* = 20**	***n* = 50**	***n* = 100**
0.5	Poi	1	1	1
0.5	ZIP	1	1	1
0.5	arcsinh	0.796	0.781	0.953
0.5	ZINB	0.498	0.498	0.499
1	Poi	1	1	1
1	ZIP	1	1	1
1	arcsinh	0.683	0.831	0.883
1	ZINB	0.497	0.501	0.499
5	Poi	0.999	1	1
5	ZIP	0.999	1	1
5	arcsinh	0.673	0.796	0.877
5	ZINB	0.526	0.513	0.509
10	Poi	0.903	0.988	1
10	ZIP	0.909	0.990	1
10	arcsinh	0.733	0.826	0.913
10	ZINB	0.562	0.526	0.515
20	Poi	0.675	0.831	0.920
20	ZIP	0.686	0.837	0.925
20	arcsinh	0.773	0.902	0.966
20	ZINB	0.628	0.564	0.548
50	Poi	0.526	0.567	0.644
50	ZIP	0.548	0.582	0.658
50	arcsinh	0.799	0.914	0.985
50	ZINB	0.742	0.667	0.512

True model: negative binomial GLMM; competing modeling strategies: Poisson, ZIP, NMM for arcsinh-transformed counts, ZINB. Sample size: **n = **20, 50, 100 patients per group; degree of overdispersion: ***k* = **0.5, 1, 5, 10, 20, 50. AUC can be interpreted as a summary measure for the goodness of discrimination between the true negative binomial model generating the longitudinal data and rival models which should be taken into consideration in practice. For ***k***→*∞*, the difference between a negative binomial and the alternative Poisson model dissolves because of convergence in distribution; therefore, AUC ***→***0.5 and both model alternatives approximate with respect to their forecasting ability.

An AUC near 1 indicates that mean log score perfectly discriminates between the true NB model and a competing (wrong) model adopted for a particular scenario. Since the true data generating distribution was negative binomial without an excess of zeros, the ZINB model did not perform worse than the true NB model and is suitable for prediction in almost the same manner, resulting in an AUC value of approximately 0.5 for all scenarios. For small *k*, the NB models clearly outperforms the competing (zero-inflated) Poisson models that do not account for overdispersion. Analyzing negative binomial data with an arcsinh NMM as an alternative to accomplish variance-stabilization, the AUC is lower than that of a wrong Poisson model. However, if *k*→*∞* and the amount of overdispersion goes down, the choice of an NMM for arcsinh-transformed counts results in AUC clearly larger than 0.5. Hence, the quality of observation-level predictions of the NMM is worse than that of the (zero-inflated) Poisson. If the negative binomial converges in distribution to the Poisson, the arcsinh-transformation of the count outcome is no longer appropriate.

## Discussion

We have discussed Bayesian strategies for model evaluation of GLMMs for longitudinal count data and used integrated nested Laplace approximations to do the calculations. We especially looked at tools such as the DIC, logarithmic score, and PIT. These techniques for model assessment are implemented in the package R‐INLA which can easily be used in R and aim to score the models with respect to their appropriateness explaining the observed data. Therefore, a very practical toolbox is at the hand for statisticians. It must be noted that other instruments such as pivotal quantities [71] or different proper scoring rules [28] can be used if the calculations are done with MCMC methods (e.g. using WinBUGS [55,72]).

We applied this toolbox to the typical task of a clinical trial statistician of making decisions for pre-specified sensitivity analyses or the efficacy analysis in a statistical analysis plan. Data from a former trial were used as pilot data for an ongoing phase III trial. Our interest was to give some insight and guidance in the most important aspect of deciding on a final SAP. The main task consisted of deciding which GLMM should be used for longitudinal count data. To this end, we performed a Bayesian analysis of the pilot data with different models and employed a prediction-based approach to derive statements on model fit.

We next discuss four important aspects of this process: prior distributions, normality assumption for random effects, Bayesian model evaluation, and modeling of clinical trial data.

### Prior distributions

Bayesian analysis needs a specification of prior distributions. However, when fitting a GLMM in a Bayesian setting, specifying prior distributions is not straightforward; this is particularly true for variance components. Fong *et al.*[40] pointed out that the priors for variance components should be chosen carefully. To quantify the sensitivity of the posterior distributions with respect to changes in the priors for the random effects precision parameters, Roos & Held [73] discuss a measure based on the so-called Hellinger distance for GLMMs with binary outcome but not for count data. Adapting their approach to count data is a topic for future research. In this study, we followed advice from the literature: in the case of negative binomial models, estimation of the posterior mean of the dispersion parameter can be affected when a vague prior specification is used to characterize the gamma hyper-parameter. To circumvent the problem of distorting posterior inferences, e.g. Lord *et al.*[74] recommend a non-vague prior distribution for the dispersion parameter to minimize the risk of a mis-estimated posterior mean and to obtain stable and valid results. This issue is particularly relevant for data characterized by a small sample size in combination with low sample mean values. The situation is quite complex and the only practical way to handle this issue is a careful simulation study to investigate whether changing priors would influence the decision on the relevant model. The material provided in the Web Supplement may help a statistician set up such simulation studies.

### Gaussian random effects

Throughout our article the distribution of random effects was assumed to be Gaussian. One reason was that Bayesian inference was based on the INLA approach. Within the INLA methodology an extension to non-Gaussian random effects is not straightforward due to the central role of the latent Gaussian field. The main challenge in applying INLA to latent models is that the approach depends heavily on the latent Gaussian prior assumption to work properly. For further details on this issue see [75]. Recently, Martins & Rue [75] proposed an extension that allows INLA to be applied to models where some independent components of the latent field have a so-called “near-Gaussian” prior distribution. All in all, the assumption of Gaussian distributed random effects that is usually taken for granted may be subject to criticism, and there are a number of situations in which this might not be a realistic assumption. From a theoretical point of view, this normality assumption may be dropped in favour of other symmetric but heavier-tailed densities, such as the Student *t*-distribution which allows to identify and accommodate for outliers both on the level of the within-group errors but also at the level of random effects [76]. Further research is needed to investigate the impact of inappropriate distributional assumptions, i.e. to understand its influence not only on posterior inference, but also on several Bayesian instruments which are applied for model evaluation.

### Bayesian model evaluation

The INLA approach for approximate fully Bayesian inference on the class of latent Gaussian models provides an attractive and convenient alternative to an inference scheme based on sampling-based methods such as MCMC, and avoids its computational burden. By taking advantage of the properties of latent Gaussian models, INLA outperforms MCMC schemes in terms of both accuracy and speed. Bayesian approaches naturally lead to posterior predictive distributions, from which any desired functional can readily be computed. We earlier discussed Bayesian methods for assessing probabilistic forecasts via proper scoring rules serving as a loss function. These scores can be used for an omnibus evaluation of both sharpness and calibration of predictive distributions and provide a usable instrument for assessing the validity of different competing, non-nested modeling strategies. It should also be noted that there is a variety of proper scoring rules with a unique and well-defined underlying decision problem which can be applied in a given situation as well. According to Gneiting [62] there are many options and considerations in choosing a specific scoring function, and there is a need for theoretically principled guidance. In this article, we have focused on the logarithmic score which is easily calculated and available from INLA. The mean logarithmic score was competitive for the simulated negative binomial data, and most importantly, it was able to identify the correct model as the one best suited for prediction, namely the true model generating the data emerged with the smallest $\left(\overset{Â¯}{\mathrm{LS}}\right)$. In contrast to proper scoring rules, PIT histograms allow evaluation of the predictive quality of a model with respect to calibration only, neglecting sharpness. Furthermore, the DIC was applied as a common model selection criteria that takes into account goodness of fit while penalizing models for overfitting. Despite its computational simplicity, DIC does have several drawbacks, particularly tending to under-penalize complex hierarchical models. Likewise, DIC is not suitable for comparing a model for transformed outcome with competing models for data on the original scale. Accordingly, predictive checks should be preferred to rank different non-nested GLMMs alternatives. Nevertheless, the question of what constitutes a noteworthy difference in DIC or mean scores to distinguish between competing model types has not yet received a satisfactory answer. For Bayes factors, calibration scales have been proposed, but no credible scale has been proposed for the difference in DIC or the difference in mean scores [54]. In conclusion, we recommend using several instruments for model evaluation to gain further insight into different aspects of a statistical model, such as forecasting ability, combined assessment of calibration and sharpness, and comparison of features of the model-based posterior predictive distribution to equivalent features of the observed data.

### Modeling of clinical trial data

In the clinical trial setting, Bayesian instruments based on the INLA approach can be applied as decision support to pre-specify a suitable final model for sensitivity analyses. Provided that adequate pilot data exist, an appropriate modeling strategy is developed using prior information obtained from a trial in an earlier phase. Sensitivity analyses are important to investigate the effects of deviations from a statistical model and its underlying assumptions. Furthermore, it is necessary to assess in what way the (posterior) inference depends on extreme observations and on unverifiable model assumptions. Altogether, situation-specific robustness of the proposed analyses must be checked carefully.

## Conclusions

The statistical model must be specified in the SAP before acquiring the real trial data. Similar independent data (for example, from patients treated with the standard treatment) may serve as a basis for decision-making. The analyses proposed in the SAP have to be appropriate and should rely on a minimum of assumptions [77,78]. Sensitivity analyses help to assess whether, for example, results from simple testing procedures applied to the primary efficacy analysis agree with the results obtained by additional, more complex analyses. These analyses may consider more complex settings for individual-level parameters, or different distributional forms of individual inhomogeneity. By studying agreement between different strategies via sensitivity analyses in the statistical report, such as simple tests for the primary analysis together with modeling approaches for sensitivity analyses, it is possible to explore robustness and accurate estimation of the treatment effects.

We look at the situation when there are various possible analyses of a given hypothesis (in our case: no treatment×time interaction), all of which have different distributional assumptions (specified by different assumptions in terms of the random effects structure and corresponding distributional assumptions). In this case, robustness would come from different analyses, with different assumptions, showing substantial agreement. On the first look, there is no ordering that would allow us to declare one analysis better than another by virtue of relying on a specific distributional assumption. On a second look, the logarithmic score, the DIC, and the PIT provide scores to establish such an ordering.

We concentrated on justifying the distributional assumptions in the count response situation, that is, checking deviations from the assumptions regarding the stochastic part of the hierarchical model, since there was no evidence for specific prognostic factors or factors for baseline adjustment, which would improve the precision of the results when considered in the model. We also explored the effect of random effects structures that include subject-specific intercepts and/or slopes. The simulation could also demonstrate how much power would be lost if we chose a more general model (NB distribution, random intercept and random slope) compared with a simple model (Poisson distribution, random intercept, no random slope) when the simple model is true.

However, the Bayesian toolbox used is all-purpose and can be applied to detect other more complex forms of misspecification as well, such as non-Gaussian distributed random effects [75], alternative functional relationships in the population-mean structure, random effects precisions depending on a binary covariate (e.g. treatment group), alternative prior distributions or different hyperprior parameter values. Finally, part of the sensitivity analyses of the trial data may also be checking whether the modeling assumptions for the primary efficacy analysis are reasonable or not.

## Appendix

### A1. Variance stabilizing transformation for negative binomial outcome

Assuming a random variable *Y*∼NB(*k*,*p*) using the notation as described before. In the limit, if dispersion parameter *k* moves to infinity, then E_*μ*_[*Y*] = Var_*μ*_[ *Y*] = *μ* and negative binomial NB(*k*,*p*) converges to Poi(*μ*) in a distributional matter. Hence, variance of *Y*∼ NB(*k*,*p*) can be described as a function of *μ*, i.e. Var_*μ*_[*Y*] = *μ*(*μ*/*k* + 1): = *v*(*μ*), for *μ*≥0.

Searching for an asymptotic variance-stabilizing function means searching for a function $\left(T:R\mapsto R\right)$ with the following property

(9) $${\text{Var}}_{\mu }[T(Y)]\approx \text{const}:=\mathrm{c.}$$

The transformation *T* is assumed to be strictly monotone, and without loss of generality it is assumed to be strictly increasing. Transformations satisfying (9) do not necessarily exist; but if they do exist, they are unique [79]. Using Taylor approximation

$$T(Y_{\mu })\approx T(\mu )+T^{\prime }(\mu )\cdot (Y_{\mu }-\mu )$$

we can write

$$\begin{array}{lll} {\text{Var}}_{\mu }[\;T(Y)\;] & \approx {\text{Var}}_{\mu }[\;T(\mu )+T^{\prime }(\mu )\cdot (Y-\mu )\;]\; & \;\\ & ={\left(T^{\prime }(\mu )\right)}^{2}\cdot {\text{Var}}_{\mu }[Y-\mu ]\; & \;\\ & ={\left(T^{\prime }(\mu )\right)}^{2}\cdot v(\mu ).\; & \; \end{array}$$

The goal is to find a real-valued, measurable transformation *T* such that $\left(1={\text{Var}}_{\mu }[\;T(Y)\;]\approx {\left(T^{\prime }(\mu )\right)}^{2}\cdot v(\mu )\right)$. This produces

#### Lemma

Let *Y*_*μ*_ be a family of random variables with mean E_*μ*_[*Y*]=*μ* and variance Var_*μ*_[*Y*]=*v*(*μ*). Then the asymptotic variance-stabilizing transformation for *Y * is given by

$$T(y)=\overset{y}{\underset{-\infty }{\int }}\frac{1}{\sqrt{v(u)}}\;\mathrm{\partial u},$$

achieving that Var_*μ*_[ *T*(*Y*) ] is independent from *μ*. □

Hence, according to this Lemma the asymptotic variance-stabilizing transformation for a negative binomial distribution is given by

(10) $$\begin{array}{cc} T(y) & =\overset{y}{\underset{0}{\int }}\frac{1}{\sqrt{v(u)}}\;\mathrm{\partial u}=\overset{y}{\underset{0}{\int }}\frac{1}{\sqrt{u(z\;u+1)}}\;\mathrm{\partial u}\\ =\text{arcsinh}(\sqrt{z\;y})/\sqrt{z} \end{array}$$

where *z*≡1/*k*represents an overdispersion parameter which is defined by specifying NB(*kp*). For *z*→0 (i.e. *k*→*∞*),

$$\text{arcsinh}(\sqrt{\left(z\;y\right)})/\sqrt{z}\;\to \;\sqrt{z}.$$

Therefore, the variance stabilizing transformation for Poisson distribution is $\left(\sqrt{z}\right)$. See [79,80] for a more detailed derivation. □

### A2. Backcalculation of the mean logarithmic score for arcsinh-transformed outcome to the original count scale

If count data exhibit overdispersion with respect to a Poisson model a *Normal* mixed effects model (NMM) for arcsinh-transformed count response can be performed to accomplish stabilization of variance. However, this involves that the predictive performance measures such as the proper scoring rules are not computed on the original scale. In this section, we detail the calculation of a “*correction term*” needed to back-transform the mean log score for the arcsinh NMM to the mean log score within the original count scale. Note that only the *mean* of log scores can be back-transformed, not the log score values of the observational level.

Let *ϕ* be the probability density for *Y * in $\left(R\right)$, and *ψ* be the probability density of *Z* in $\left(R\right)$, with $\left(z=g(y)=\text{arcsinh}(y)=\log(y+\sqrt{y^{2}+1})\right)$, $\left(g^{-1}(z)=\frac{\exp(z)-\exp(-z)}{2}\right)$, with the derivation $\left(g^{\prime }(y)=\frac{1}{\sqrt{1+y^{2}}}\right)$.

Suppose we have computed the mean log score, denoted as mean(LS) | _arcsinh_. Using the substitution *y*=*g*^−1^(*z*) and $\left(\mathrm{dy}={\left[g^{-1}\right]}^{\prime }(z)\;\mathrm{dz}\right)$, the following equations hold:

(11) $$\begin{array}{lll} & \text{mean(LS)}\;|{\;}_{\text{original}}\; & \;\\ & \;=\;E\;\left[-\log(\phi (Y))\right]\; & \;\\ & \;=\;\overset{\infty }{\underset{-\infty }{\int }}[-\log\phi (Y)]\cdot \phi (Y)\;\mathrm{dy}\;\\ & \;=\;\overset{\infty }{\underset{-\infty }{\int }}[-\log\phi (g^{-1}(z))]\cdot \phi (g^{-1}(z))\cdot {\left[g^{-1}\right]}^{\prime }(z)\;\mathrm{dz}\; & \;\\ & \;=\;\overset{\infty }{\underset{-\infty }{\int }}\left[-\log\phi (g^{-1}(z))\right]\cdot \frac{\phi (g^{-1}(z))}{g^{\prime }[g^{-1}(z)]}\;\mathrm{dz}\; & \;\\ & \;=\;\overset{\infty }{\underset{-\infty }{\int }}\left[-\log\left\{\psi (z)\cdot g^{\prime }[g^{-1}(z)]\right\}\right]\psi (z)\;\mathrm{dz}\; & \;\\ & \;=\;\underset{\text{mean(LS)}\;|{\;}_{\text{arcsinh}}}{\underset{︸}{E\;\left[-\log\psi (Z)\;\right]}}-\;E\;\left[\log\{g^{\prime }[g^{-1}(Z)]\}\;\right]\; \end{array}$$

where $\left({\left[g^{-1}\right]}^{\prime }(z)=\frac{1}{g^{\prime }[g^{-1}(z)]}\right)$ and $\left(\psi (z)=\frac{\phi (g^{-1}(z))}{g^{\prime }[g^{-1}(z)]}\right)$. Hence, the correcting subtrahend required to convert the mean log score for the arcsinh NMM to the original scale results from

$$E\;\left[\log\{g^{\prime }[g^{-1}(Z)]\}\;\right].$$

This is simply the empirical sample mean of a certain transformation of the materialized and the arcsinh-transformed counts, respectively. After backcalculation the mean log score for the arcsinh NMM can be compared with the mean log scores of other model alternatives assessing their forecasting capability.

### A3. Vertigo data: comparison of INLA and MCMC

Figure 2 illustrates the approximated posterior marginals for the fixed effects (posterior marginal distribution for intercept and hyperparameters not shown). The dotted curve with overlaid histogram is the posterior marginal density resulting from a MCMC run based on 1000 (near independent) samples; the output was constructed with the built-in MCMC-sampler (more precisely the “one-block MCMC-sampler” described in [81, chapter 4]) available within the inla program). Apparently, the Laplace approximation gives an accurate fit indicating that MCMC and INLA provide comparable posterior estimates in this longitudinal setting.

## Competing interests

The authors declare that they have no competing interests.

## Authors contributions

The authors’ responsibilities were as follows: CA (guarantor) did the statistical analysis, designed the simulation study and wrote the first draft of the manuscript. UM reviewed and critiqued the manuscript and made substantial contributions to subsequent drafts. All authors checked and approved the final version of the manuscript.

## Pre-publication history

The pre-publication history for this paper can be accessed here:

http://www.biomedcentral.com/1471-2288/12/137/prepub

## Supplementary Material

Additional file 1 **Web-based Supplementary Material.** This document contains further technical details concerning the INLA approach. Furthermore, chunks of R code to illustrate the use of the R package R‐INLA are provided. Click here for file

Additional file 2 **Rfile to generate NB GLMM data used in the simulation study.**R function make.negbin.rfc(.) creates a data set with longitudinal counts (data generating process used: negative binomial random intercept models for varying degrees of overdispersion and sample sizes). Click here for file
