# Supplementary material for: Bayesian model selection techniques as decision support for shaping a statistical analysis plan of a clinical trial: An example from a vertigo phase III study with longitudinal count data as primary endpoint
Source: BMC Med Res Methodol. 2012 Sep 10;12:137. doi: 10.1186/1471-2288-12-137 (PMC3554595; doi:10.1186/1471-2288-12-137)
Supplement: Additional file 1 — Web-based Supplementary Material. This document contains further technical details concerning the INLA approach. Furthermore, chunks of R code to illustrate the use of the R package R‐INLA are provided. [file 1471-2288-12-137-S1.pdf]

Web-based Supplementary Material

Bayesian model selection techniques as decision support for shaping a statistical analysis plan of a clinical trial: An example from a vertigo Phase III study with longitudinal count data as primary endpoint

Christine Adrion, Ulrich Mansmann

0 Introduction

The software `R-INLA` (more precisely: the program `inla` bundled within an R interface) was created to provide a user-friendly tool for performing approximate Bayesian inference on a range of *latent Gaussian models* using integrated nested Laplace approximations (INLAs) [1, 2, 3]. Latent Gaussian models include, e.g., (generalized) linear (mixed) models, (generalized) additive models, smoothing spline models, state space models, semiparametric regression, spatial and spatio-temporal models, or geostatistical models.

The INLA computational approach combines Laplace approximations and numerical integration in a very efficient matter (using sparse matrix algorithms), without resorting to MCMC techniques. INLA substitutes MCMC simulations with very accurate deterministic approximations of the posterior marginal distributions. In addition to its computational speed, latent Gaussian models are treated in an unified way, and therefore, allowing greater automation of the inference process. For a detailed description of the INLA methodology as well as a thorough comparison with MCMC results please refer to [1].

The output of `inla` consists of approximate posterior marginal distributions, which can be used to compute summary statistics of interest, such as posterior means, variances or quantiles. Furthermore, the DIC, PIT and CPO values, or the logarithmic scores can be obtained to compare and assess complex Bayesian hierarchical models.

The Website `r-inla.org` includes a short documentation describing the class of hierarchical models which can be solved within the `R-INLA` library, and for each model a detailed description and an example of usage is provided. The latest release of `R-INLA` to implement the INLA approach can also be found at this site. Currently, the R-library implements many exponential family models, e.g., Gaussian, Poisson, Binomial, negative binomial, zero-inflated extensions, etc. Dependence can be modelled using, e.g., random effects, first order auto-regressive, first and second order random walks, and much more.

1 Web Appendix: Implementing GLMMs in INLA

In the following it is demonstrated how generalized linear mixed effects models used in the Results Section (Simulation Study) are coded in `R-INLA`. In this supplement we focus on *random intercept models* and provide a description of selected R-code required for the implementation of the INLA approach.

In `R-INLA`, a random intercept model is constructed in 2 steps:

- Specification of the latent Gaussian field through the `formula` mechanism, by using the `f()` function to define the type of latent Gaussian field, e.g., random intercepts denoted as 'independent random noise model' (`model="iid"`)
- `inla()`-call

Within the `inla()`-call many further options and additional features for the INLA algorithm can be set. Particularly, the flags `cpo=TRUE` or `dic=TRUE` tell the `inla()` function to compute "leave-one-out" predictive measures, namely CPO and PIT values, or the DIC. For computation of CPO and PIT quantities, it is recommended to increase the accuracy of the tails of the marginals, by modifying the `control.inla` argument: For instance, we chose the full Laplace approximation (`strategy="Laplace"`), added more evaluation points (`npoints=...`) or changed the integration strategy (`int.strategy="grid"`). The default choice is the simplified Laplace approximation and the so-called central composite design (CCD) integration scheme [1, 3].

The dataframe to be used is specified using the `data` argument within the `inla()`-call. The prior for the hyperparameter (i.e. the precision parameter) of a single random effect is specified inside the `f()` function (using `prior=...` and `param=...` statements to change default hyperprior distributions and corresponding hyperprior parameters). If a prior for the hyperparameter of an observational model has to be specified, e.g., for the dispersion parameter k in case of a negative binomial model or for the precision in case of a Normal response model, the prior has to be assigned inside the `control.data` argument.

Beware that INLA is still a project under active development – hence, parts of the following `R-INLA` code may have changed in the meanwhile. For the analyses within this paper we used the `R-INLA` library built in October 2011.

Simulation scenario for longitudinal negative binomial counts:

Example: number of patients per group: $n = 20$, overdispersion parameter $k = 1$.

Install the `R-INLA` software by typing the following command line in R:

```
> source("http://www.math.ntnu.no/inla/givemeINLA.R")
> require(INLA)           # load the INLA library

> #--- simulate longitudinal negative binomial counts:
> #    data generating process: random intercept model
> #                    mu(t) = exp{a + a.i + t*[gg*b.2 + (1-gg)*b.1]}
>
> source("./neg.binom.rfc.r")  # load function 'make.negbin.rfc(.)'
>
> # resulting dataset: subject (sub), group, time (tt), counts (y)
> #
> # Example:
> # set.seed(123)
> # dd = make.negbin.rfc(n = 20, size = 1)
> # head(dd)
> #   sub group tt  y
> # 1   1     0  0  3
> # 2   1     0  1  0
> # 3   1     0  2 11
> # 4   1     0  3  4
> # 5   2     0  0 22
> # 6   2     0  1  9
> #    ...   ...
```

Chunk of R-code to perform full Bayesian inference on a negative binomial random intercept model:

```
> #==========================================================================#
> # Negative binomial random intercept model:
> #==========================================================================#
> # Note: for simplicity, only 1 Run is displayed here...
>
> set.seed(123)
> dd = NULL
> m.nb = NULL
> erg = NULL
>   dd = make.negbin.rfc(n = 20, size = 1)
>   sub2 = dd$sub
>   # specifying the 'independent random noice model' named "iid" inside the f(.)-function,
>   # as well as the hyperparameters for hyperprior of precision
>   formula = y ~ tt + group:tt +  f(sub, model = "iid", param=c(0.5, 0.0011))
>   m.nb <- inla.cpo(formula = formula, family="nbinomial", data = dd,
+                 control.inla=list(strategy = "laplace", int.strategy = "grid",
+                                     diff.logdens = 4, npoints = 21),
+                 control.fixed = list(prec.intercept = 0.0001, prec = 0.001),
+                 control.compute = list(dic = TRUE, cpo = TRUE, mlik =T),
+           control.data = list(hyper = list(theta = list( prior="gaussian", param = c(0,0.01)))))
>                  #prior specification for overdispersion parameter by using the statement hyper=...
>
```

```
>   erg = list(cpo = m.nb$cpo$cpo,
+              pit = m.nb$cpo$pit,
+              dicall = m.nb$dic, dic = m.nb$dic$dic,
+              ls = mean(-log(m.nb$cpo$cpo))
+              )
> erg$dic  # DIC
> # [1] 1168.65
>
> erg$ls   # mean logarithmic score
> #[1] 3.65336
```

Chunk of R-code to perform full Bayesian inference on a Poisson random intercept model:

```
> #===============================================================================#
> # Poisson random intercept model [100 Runs]:
> #===============================================================================#
>
> set.seed(123)
> dd = NULL
> m.poi = NULL
> erg = NULL
> for (i in 1:100)
+ {
+   dd[[i]] = make.negbin.rfc(n = 20, size = 1)
+
+   sub2 = dd[[i]]$sub
+
+   formula = y ~ tt + group:tt +  f(sub, model = "iid", param=c(0.5, 0.0011))
+
+   m.poi[[i]] <- inla.cpo(formula=formula, family="poisson", data = dd[i]],
+                    control.inla = list(strategy = "laplace", int.strategy = "grid"),
+                    control.fixed = list(cdf = c(0)),
+                    control.compute = list(dic = TRUE, cpo = TRUE, mlik =T))
+
+   erg[[i]] = list(cpo = m.poi[[i]]$cpo$cpo,
+                   pit = m.poi[[i]]$cpo$pit,
+                   dicall = m.poi[[i]]$dic, dic = m.poi[[i]]$dic$dic,
+                   ls = mean(-log(m.poi[[i]]$cpo$cpo))
+                  )
+
+ }
```

Chunk of R-code to perform full Bayesian inference on a normal random intercept model – after the variance-stabilizing transformation of the count response data was applied.
Note: The resulting mean logarithmic score is computed on the transformed scale, and has to be back-calculated to the original count scale before model comparison! (see Appendix A.2 of our article)

```
> #===============================================================================#
> # NMM for arcussinh-transformed count outcome [100 Runs]:
> #===============================================================================#
> set.seed(123)
> dd = NULL
> m.asinh = NULL
> erg = NULL
> for (i in 1:100)
+ {
+   dd[[i]] = make.negbin.rfc(n = 20, size = 1)
+
+   sub2 = dd[[i]]$sub
+
+   formula.RS = asinh(y) ~ tt + group:tt +  f(sub, model = "iid", param=c(0.5, 0.0011))
+
+   m.asinh[[i]] <- inla.cpo(formula=formula.RS, family="normal", data = dd[[i]],
+                     control.inla=list(strategy = "laplace", int.strategy = "grid"),
+                     control.fixed = list(cdf = c(0)),
+                     control.compute = list(dic = TRUE, cpo = TRUE, mlik =T), verbose = T)
+
```

```
+   erg[[i]] = list(cpo = m.asinh[[i]]$cpo$cpo,
+                   pit = m.asinh[[i]]$cpo$pit,
+                   dicall = m.asinh[[i]]$dic, dic = m.asinh[[i]]$dic$dic ,
+                   ls = mean(-log(m.asinh[[i]]$cpo$cpo))
+                  )
+ }
```

Zero-inflated extensions of Poisson or negative binomial mixed models are specificed by `family="zeroinflatednbinomial1"` and `family="zeroinflatedpoisson1"` respectively, resulting in an additional zero-inflation hyperparameter π_0 within the observational model. with associated prior distribution.

References

[1] Rue H, Martino S, Chopin N: Approximate Bayesian inference for latent Gaussian models by using integrated nested Laplace approximations. *Journal of the Royal Statistical Society: Series B (Statistical Methodology)* 2009, **71**(2):319–392.

[2] Simpson D, Lindgren F, Rue H: Fast approximate inference with INLA: the past, the present and the future. Department of Mathematical Sciences, NTNU, Trondheim, Norway 2011, [http://arxiv.org/pdf/1105.2982v1]. [Technical report at arxiv.org].

[3] Martino S, Rue H. Case Studies in Bayesian Computation using INLA. In Complex Data Modeling and Computationally Intensive Statistical Methods. Edited by Mantovan P, Secchi P, Milan: Springer Verlag Italia 2010:99–114.

[4] Website www.r-inla.org, see e.g., a short introduction available at http://www.math.ntnu.no/~hrue/r-inla.org/doc/Intro/Intro.pdf
